# Sustainable Lipase Immobilization: Chokeberry and Apple Waste as Carriers

**DOI:** 10.3390/biom14121564

**Published:** 2024-12-08

**Authors:** Karina Jasińska, Maksym Nowosad, Aleksander Perzyna, Andrzej Bielacki, Stanisław Dziwiński, Bartłomiej Zieniuk, Agata Fabiszewska

**Affiliations:** 1 Department of Chemistry, Institute of Food Sciences, Warsaw University of Life Sciences-SGGW (WULS-SGGW), 159c Nowoursynowska St., 02-776 Warsaw, Poland; agata_fabiszewska@sggw.edu.pl; 2 Department of Food Biotechnology and Microbiology, Institute of Food Sciences, Warsaw University of Life Sciences-SGGW (WULS-SGGW), 159c Nowoursynowska St., 02-776 Warsaw, Poland; maksym_nowosad@sggw.edu.pl; 3 Faculty of Biology and Biotechnology, Warsaw University of Life Sciences—SGGW, 159 Nowoursynowska Street, 02-776 Warsaw, Poland; alek_2000-00@wp.pl (A.P.); andrzej.bielacki2@onet.pl (A.B.); stanislaw.dziw@gmail.com (S.D.)

**Keywords:** lipase, immobilization, apple pomace, chokeberry pomace

## Abstract

In the modern world, the principles of the bioeconomy are becoming increasingly important. Recycling and reusability play a crucial role in sustainable development. Green chemistry is based on enzymes, but immobilized biocatalysts are still often designed with synthetic polymers. Insoluble carriers for immobilized biocatalysts, particularly those derived from agro-industrial waste such as mesoporous lignocellulosic materials, offer a promising alternative. By using waste materials as support for enzymes, we can reduce the environmental impact of waste disposal and contribute to the development of efficient bioprocessing technologies. The current study aimed to assess the possibility of using apple and chokeberry pomace as carriers for the immobilization of Palatase 20000L (lipase from *Rhizomucor miehei*). The analysis of lignocellulosic materials revealed that chokeberry pomace has a higher neutral detergent fiber (NDF) and lignin contents than apple pomace. Moreover, Scanning Electron Microscopy (SEM) observations indicated similar compact structures in both pomaces. The lipase activity assays demonstrated that immobilization of lipase from *R. miehei* onto apple and chokeberry pomace improves their properties, especially the synthetic activity. The findings highlight the potential of utilizing fruit pomaces not only as a source of bioactive compounds but also in enhancing enzyme stability for industrial applications.

## 1. Introduction

Climate change and environmental degradation pose a serious threat to East European countries and the world as a whole. Waste processing and landfilling are contributing factors. The global food supply chain is one of the most complex and interconnected systems worldwide and one of the least sustainable [1]. The European Union, as part of the European Green Deal, aims to achieve zero emissions and maintain clean soils and waters to mitigate climate change. Therefore, it is crucial to explore new green and cost-effective solutions. One approach that aligns with these objectives is upcycling, which involves repurposing discarded materials to increase their value [2]. In fruit and vegetable processing, a significant problem is the disposal of products that are not used in the technological process. Pomace accounts for 10 to 35% of processed raw materials [3]. This problem is particularly relevant for countries such as Poland, one of the leading producers of fruits such as apples, pears, and chokeberries, which are used to produce juices, jams, or concentrates. According to the Central Statistical Office in Poland, in the 2018/2019 season, only 3150 thousand tons of apples were used to produce concentrated juice [4]. In numerous countries, apple pomace (AP) is one of the most frequently produced types of agri-food waste, with about 4 million tons being produced each year worldwide [5]. According to Poland’s Agricultural Market Agency, the area of chokeberry fields in Poland increased from 5000 hectares to 8000 hectares between 2004 and 2013, with the yield rising from 38,000 to 58,000 metric tons [6].

The growing interest in sustainable practices has led to increased efforts in finding effective ways to repurpose lignocellulosic materials, including apple and chokeberry waste, from juice and jam manufacturing. Waste materials from the fruit and vegetable industry with adequate porosity, surface charge, and inertness to the product can serve as effective carriers in the immobilization process [7,8]. Over the years, scientists have tested many waste products as suitable carriers, i.e., spent grain after beer production, spent coffee grounds, cashew apple and sugarcane bagasse, and coconut waste, some of which have translated into providing better yields of the enzyme used [9,10,11,12,13].

Many types of enzymes are used in biotechnology and industry, such as hydrolases, lipases, and peptidases. Lipases, also known as triacylglycerol acylhydrolases (EC 3.1.1.3.), are a group of enzymes that can act on insoluble substrates emulsified in water and catalyze the hydrolysis of long-chain fatty acids in triacylglycerides. Lipases differ from esterases, which act on water-soluble substrates like simple esters with short-chain fatty acids. Furthermore, they are the third largest family of digestive enzymes, following proteases and carbohydrates, and the main group of biocatalysts in the biotechnology industry. In addition to their hydrolytic activity on triacylglycerides, lipases catalyze reverse reactions in non-aqueous media, such as esterification and transesterification [14].

The lipase from *Rhizomucor miehei* (RML) is a commercially available enzyme in free and immobilized forms [15]. An important issue from an industrial perspective is the insufficient activity and stability of free enzymes [16]. Researchers have discovered that immobilization is an effective solution to these challenges. The functions and properties of the immobilized enzyme depend on the type of protein used, as well as the immobilization technique and the material used as a carrier [17]. An ideal immobilization method should not only provide high activity and stability for the enzyme but also maintain a good balance between performance and price, which is particularly important in large-scale production [18]. The method of enzyme immobilization can be based on chemical reactions or physical adsorption. Adsorption relies on the physical interactions between the carrier and enzyme, such as van der Waals forces, ionic interactions, and hydrogen bonding. These interactions are relatively weak and, importantly, typically do not alter the native structure of the enzyme. This helps to maintain the enzyme’s active sites and allows it to retain its activity [19]. 

In the current study, the main goal was to answer whether chokeberry and apple pomace could be effective carriers for enzyme immobilization. The purpose of the work was also to see if this opens up new avenues for the use of food waste in the field of biotechnology with simultaneous recycling and finding new methods of environmental protection and whether this will allow a change in the approach to enzyme immobilization, combining new ecological outputs and saving finances spent on immobilization. So far, several studies have examined food waste as carriers for immobilizing lipolytic enzymes, such as eggshells, spent coffee grounds, or brown onion skins [20]. Therefore, to the authors’ knowledge, fruit pomaces have never been utilized in the immobilization process. 

## 2. Materials and Methods

### 2.1. Materials and Biocatalyst

The present study used apple and chokeberry pomaces (Greenherb Company, Wysoka, Poland) as a support for lipase immobilization. Liquid lipase Palatase 20000L (from *Rhizomucor miehei*), kindly gifted from Novozymes (Basgvaerd, Denmark), was used as a biocatalyst. All chemical reagents and solvents were purchased from Sigma-Aldrich (Poznań, Poland). 

### 2.2. Pretreatment of Apple and Chokeberry Pomace

Three different types of matrices were used for lipase immobilization. The first was a native carrier without any pretreatment, the second involved pretreatment through hexane extraction of pomace, and the third used a hexane-ethanol extraction pretreatment of pomace. The obtained apple and chokeberry pomaces were purified of polyphenols, lipids, and other polar and non-polar substances using a Soxhlet apparatus, with a pomace-to-solvent ratio of 1:15 (*w*/*v*). The solvents employed for this process were n-hexane and ethanol, and the extraction was performed until each solvent had cycled through the system 12 times.

### 2.3. Determination of Fiber of Apple and Chokeberry Pomace

The fiber content was analyzed using the FibertecMC 8000 system (Foss Analytics, Warsaw, Poland). The percentage of crude fiber was measured using the PN-ISO 5498 [21] method. The content of acid detergent fiber (ADF) and acid detergent lignin (ADL) was assessed according to the PN-EN ISO 13906 standards [22]. The neutral detergent fiber (NDF) content, following amylase treatment, was determined based on PN-EN ISO 16472 standards. [23]. The concentration of cellulose was calculated as the difference between ADF and ADL, while the concentration of hemicellulose was calculated as the difference between NDF and ADF.

### 2.4. Lipase Immobilization Procedure

Based on the methodology described by Jasinska et al. [24], lipase was immobilized by the adsorption method onto native and pretreated apple and chokeberry pomace. Then, 1 mL of lipase (Palatase 20000L) was dissolved in 14 mL of distilled water, added to 1 g of support in a flask, and stirred for two hours. After this time, the biocatalyst was filtered using a vacuum, washed with distilled water, and dried under room conditions. After immobilizing the lipase onto the carrier, the material was stored in vials at room temperature and used for further analyses.

### 2.5. Lipase Activity Assay

#### 2.5.1. Hydrolytic Activity

The measurements were conducted using a spectrophotometric technique based on the hydrolysis of *p*-nitrophenyl laurate. The reaction was executed in Eppendorf test tubes. A total of 100 µL of free liquid lipase or 25 mg of immobilized lipase in 100 µL of distilled water was mixed at 37 °C with 25 µL of 0.3 mmol *p*-nitrophenyl laurate dissolved in 2 mL of heptane. After an incubation period of 15 min, the absorbance was promptly measured at 410 nm using a UV–Vis spectrophotometer. The enzymatic activity of lipase was defined as 1 U, meaning the quantity of enzyme that produced 1 µmol of *p*-nitrophenol per minute under the specified assay conditions.

#### 2.5.2. Synthetic Activity

The colorimetric method outlined by Jasińska et al. [24] was used to assess the synthetic activity of free and immobilized lipase. The transesterification process occurred in Eppendorf test tubes filled with 100 mM vinyl acetate, 100 mM n-butanol, and 1 mL of n-hexane. Subsequently, 10 µL of free liquid lipase or 5 mg of immobilized lipase was introduced. After incubating for 5 min, diluted samples were prepared in test tubes. To each of these samples, 1 mL of a 0.1% (*m*/*v*) MBTH (3-methyl-2-benzothiazolinone hydrazone) solution was added and stirred for 10 min at 30 °C. Then, 0.4 mL of a 1% (*m*/*v*) H_4_FeNO_4_S_2_ × 12H_2_O solution was incorporated and mixed for 30 min at 30 °C. Ultimately, the analysis of released acetaldehyde, which was converted into a blue-colored tetraaza-pentamethincyanine (TAPMC), was performed using spectrophotometric measurement at a wavelength of 595 nm.

### 2.6. Substrate Specificity

A spectrophotometric method (described in Section 2.5.1) was used to test the enzyme’s substrate specificity. Four esters were used as substrates: *p*-nitrophenyl butyrate (C4:0), *p*-nitrophenyl laurate (C12:0), *p*-nitrophenyl palmitate (C16:0), and *p*-nitrophenyl oleate (C18:1).

### 2.7. Immobilized Lipases Recovery

An evaluation of the biocatalyst’s ability to be reused was performed using a spectrophotometric technique focused on the hydrolytic activity of the samples (as described in Section 2.5.1). A single sample was utilized to hydrolyze *p*-nitrophenyl laurate in five separate instances. Following each cycle, the biocatalyst was extracted from the reaction mixture and rinsed with n-hexane and subsequently phosphate buffer (pH = 7). The prepared biocatalyst was then employed for the subsequent hydrolysis cycle.

### 2.8. Profile of pH Activity

To assess the stability of the enzyme used in the study under different pH conditions, spectrophotometric measurements of the *p*-nitrophenyl laurate hydrolysis reaction in of various solutions were conducted. Four phosphate buffers (10 mM) were prepared at pH levels 5.8, 6, 7, and 8. Solutions of pH 5 (buffer pH was lowered with hydrochloric acid), 6, 7, and 8 were used for analysis.

### 2.9. Protein Content

The concentration of protein in free liquid lipase and the filtrates following immobilization was determined using Lowry’s method [25], which involves a reaction between peptide bonds and aromatic amino acids with the Folin–Ciocâlteu phenol reagent. In this study, each examined solution comprised 1 mL, and samples were diluted fiftyfold. The reaction was conducted in test tubes by adding 5 mL of copper reagent (composed of 2% Na_2_CO_3_ in 0.1 M NaOH, 1% CuSO_4_, and 2% potassium sodium tartrate in a 100:1:1 ratio). After 10 min, 0.5 mL of the Folin–Ciocâlteu phenol reagent was incorporated. The mixture was incubated for 30 min, after which measurements were performed at 750 nm using a Rayleigh UV-1601 spectrophotometer (BRAIC, Beijing, China). A calibration curve created with albumin as a standard was employed to determine the protein content.

### 2.10. Hydrolytic and Synthetic Specific Activity

Based on the obtained results of hydrolytic and synthetic activity and protein content, the specific activities of immobilized biocatalysts and free lipase were calculated using the provided equations:

1. Liquid, free lipase:


(1) $$Specific\;activity\left[\frac{U}{\mathrm{mg}}\right]=\frac{hydrolytic\;or\;synthetic\;activity[\frac{U}{\mathrm{mL}}]}{protein\;content\;in\;liquid,\;free\;lipase\;[\frac{\mathrm{mg}\;protein}{\mathrm{mL}}]}$$

2. Immobilized lipase:


(2) $$Specific\;activity\left[\frac{U}{\mathrm{mg}}\right]=\frac{hydrolytic\;or\;synthetic\;activity\;[\frac{U}{\mathrm{mg}}]}{protein\;immobilized\;on\;support\;[\frac{\mathrm{mg}\;protein}{\mathrm{mg}\;support}]}$$

### 2.11. Scanning Electron Microscopy (SEM)

The native supports and immobilized enzyme preparations were examined for their surface structure using an electron microscope (HITACHI TM 3000, Ramsey, NJ, USA). The samples were dried in a vacuum, coated with gold layers (Cressington Sputter Coater 108 auto, Cressington Scientific Instruments, Watford, UK), and subsequently observed. Microphotographs were captured at magnifications of 200× and 600×.

### 2.12. Statistical Analysis

The outcomes were examined using the STATISTICA 13.3 software (StatSoft, Kraków, Poland). The analysis involved the following methods: the Shapiro–Wilk test to assess the normality of the data, Levene’s and Brown-Forsythe tests to evaluate the equality of variances, analysis of variance (ANOVA), and the post hoc Tukey’s test. A *p*-value of ≤0.05 was considered statistically significant.

## 3. Results and Discussion

### 3.1. Characterization of Support for Immobilization

After the juice extraction and cider production process, approximately a quarter of the fresh fruit weight is left as apple pomace. This pomace comprises residual flesh, peels, seeds, and stems from various apple cultivars. Despite being naturally considered waste, these residual materials are rich in carbohydrates, proteins, vitamins, and minerals. They also serve as a valuable source of natural antioxidants and pectins [26,27]. Chokeberry pomace is also distinguished by its composition. The residue from juice production contains significant amounts of dietary fiber, polyphenols, anthocyanins, and pectins [28].

Quantifying the amount of lignocellulosic material in carrier samples is essential due to its potential impact on immobilization. The neutral detergent fiber (NDF) was used to measure all components of plant cell walls, such as cellulose, hemicellulose, and lignin. Based on the obtained results (Table 1) for native apple and chokeberry pomace, the differences between those two sources were noticeable. The NDF value for native chokeberry residuals (55.16%) was higher than for apple waste matrices (36.32%). Chokeberry pomace had lignin, cellulose, and hemicellulose contents of 32.76%, 18.87%, and 3.53%, respectively, which were lower in comparison to data presented by Nawirska and Kwaśniewska [29], whereas cellulose (34.6%) and hemicellulose (33.5%) occurred in the most significant amounts while lignin content (24.1%) was lower. In apple pomace, lignin, cellulose, and hemicellulose contents were obtained at 9.46%, 20.99%, and 5.87%. At the same time, other researchers found lignin, cellulose, and hemicellulose contents of 18.92%, 11.56%, and 10.0% [27], or 8.87%, 16.44%, and 4.09% observed by Wang and Thomas [30], as well as 20.4%, 43.6%, and 24.4% [29], while in the study of Gullón et al. [31], these values were in the ranges 13.8–17.1%, 20.2–26.4%, and 20.0–29.9%, respectively, when nine samples of apple pomace were compared. The fruit pomace lignocellulosic content analysis yielded varying results compared to prior studies, suggesting the heterogeneous nature and diversity of apple and chokeberry byproducts.

**Figure 1:**
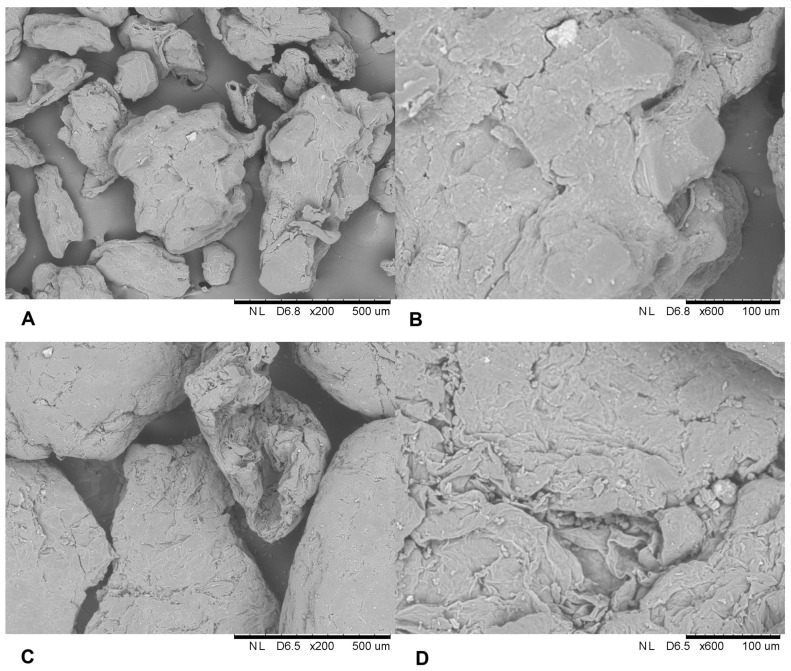
Scanning electron microphotographs (×200 and ×600) of (**A**,**B**)—native chokeberry pomace and (**C**,**D**)—native apple pomace.

The morphological structures of native carriers were analyzed using a Scanning Electron Microscope (Figure 1). It was observed that the surface of both waste matrices is similar and forms a compact structure, flattened with some visible gaps in between. Both carriers were not highly porous and did not contain loose spaces in the structure. In the case of apple pomace, fibers were more visible and arranged in layers. This structure increases the likelihood of the enzyme adsorbing onto the surface of the carrier rather than entering the matrix. 

### 3.2. Evaluation of Lipase Activity

RML (Lipase from *R. miehei*) is a type of lipase initially applied and primarily manufactured to modify oils and fats. Its use in the industry is attributed mainly to the enzyme’s high stability in anhydrous systems, which provides advantages compared to other lipases. These advantages are further supported by its high esterification activity in anhydrous environments. Consequently, RML is often the preferred lipase for esterification reactions or any process involving esterification in the initial stages (such as acidolysis and interesterification) [15].

**Figure 2:**
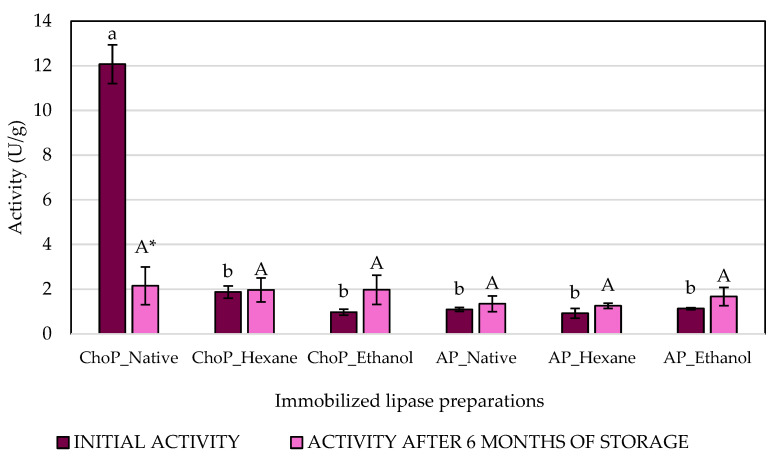
The hydrolytic activities of immobilized lipase onto chokeberry pomace—ChoP (native, after hexane and ethanol treatment) and apple pomace—AP (native, after hexane and ethanol treatment, measured initially (dark magenta bars) and after 6 months of storage (violet bars). Means with the same letter a or b for initial activity and A for activity after 6 months of storage did not differ significantly (α = 0.05). Means with * within one immobilized lipase preparation differ significantly (α = 0.05).

**Figure 3:**
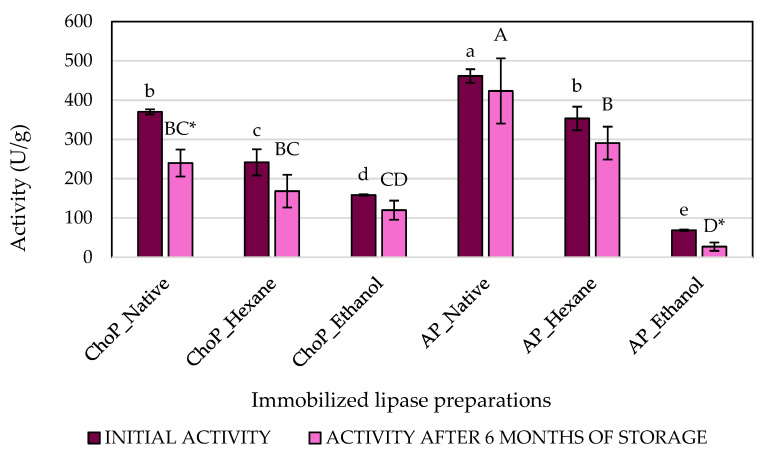
The synthetic activities of immobilized lipase onto chokeberry pomace—ChoP (native, after hexane and ethanol treatment) and apple pomace—AP (native, after hexane and ethanol treatment, measured initially (dark magenta bars) and after 6 months of storage (violet bars). Means with the same letter (a–e) or (A–D) did not differ significantly. Means with * within one immobilized lipase preparation differ significantly (α = 0.05).

The primary function of lipases is to break down oils and fats through hydrolysis. Triglycerides are molecules that are not very soluble in water. However, lipases can carry out this function, which sets them apart from standard esterases. Lipases are considered interfacial enzymes [32]. The current research analyzed the ability of lipases to catalyze hydrolysis and transesterification reactions under appropriate conditions. The lipolytic activities of lipase immobilized onto chokeberry and apple pomace after different pre-treatment methods (Figure 2 and Figure 3) were compared. Additionally, the effect of storage on both activities was investigated.

The highest hydrolytic activity was obtained for biocatalysts immobilized onto native chokeberry pomace (12.07 U/g), but after 6 months of storage, it sharply decreased (2.15 U/g). However, after storage, lipases immobilized onto chokeberry pomace pretreated with ethanol and hexane did not significantly change their activity. The hydrolytic activity of enzymes immobilized onto native apple pomace and carriers after solvent pretreatments showed no statistically significant differences between preparations and after storage. It was also noticed that the immobilization process onto apple pomace, regardless of pretreatment of the carrier, positively affects the stability of lipase hydrolytic activity over time. The same conclusion can be made for biocatalysts immobilized onto chokeberry pomace after hexane and ethanol pretreatment. This indicates that although the highest hydrolytic activity was achieved by the enzyme preparation immobilized onto native chokeberry pomace, unfortunately, this result did not persist after storage. It may seem that the compounds in the native waste had an inhibitory effect on the enzyme activity during storage, which was no longer observed for the purified pomace [33].

However, different observations were made regarding the synthetic activity of the biocatalysts. Enzyme preparations immobilized onto native apple and chokeberry pomace received the highest synthetic activities, i.e., 461 and 370 U/g, respectively. It was also observed that synthetic activity decreased with the degree of fruit waste pretreatment. Comparing the two carriers with each other, the biocatalyst immobilized onto native apple pomace had higher synthetic activity than that immobilized onto native chokeberry pomace. After 6 months of storage, lipase preparations immobilized onto chokeberry pomace after hexane and ethanol modification and onto native apple pomace after hexane pretreatment were still equally active. The only notable differences were in the biocatalyst adsorbed on native chokeberry pomace and apple pomace following ethanol pretreatment. The results indicate that the immobilization process generally ensures the stability of most biocatalysts after storage, except for the two variants mentioned earlier.

In another study, where researchers were using spent coffee grounds as carriers for the immobilization process of lipases, there was a conclusion that lipolytic enzyme preparations demonstrated the highest synthetic activity immobilized onto native spent coffee grounds, while the purification process using hexane or ethanol resulted in lower enzyme activity. The research indicated minimal variations in the hydrolytic activity of enzymes that were adsorbed on native compared to defatted forms or between defatted forms and those free of polyphenols [34].

**Figure 4:**
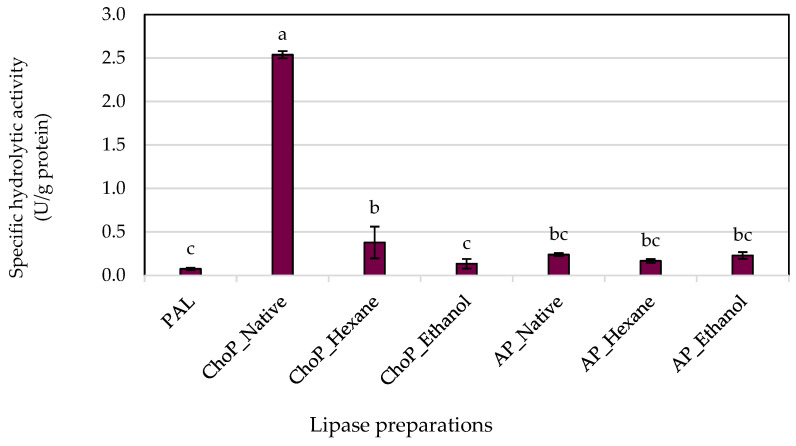
The specific hydrolytic activities of Palatase 20000L in the free form (PAL) and immobilized onto different carrier forms. Means with the same letter (a–c) did not differ significantly (α = 0.05). Abbreviations: ChoP—chokeberry pomace, AP—apple pomace.

**Figure 5:**
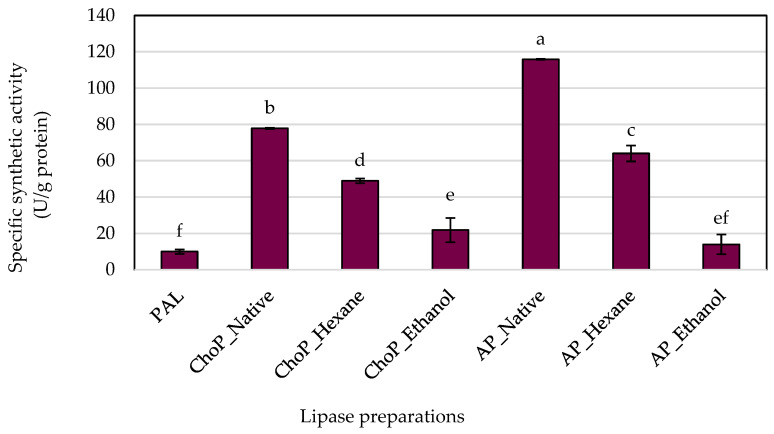
The specific synthetic activities of Palatase 20000L in the free form (PAL) and immobilized onto different carrier forms. Means with the same letter (a–f) did not differ significantly (α = 0.05). Abbreviations: ChoP—chokeberry pomace, AP—apple pomace.

The process of immobilization enhances the catalytic features of enzymes, a concept that was also explored in this study. Figure 4 and Figure 5 illustrate the effect of physically adsorbed microbial lipase on various supports regarding lipolytic activity when compared to non-immobilized enzymes. The results for both hydrolytic and synthetic activities were determined based on the protein concentrations adsorbed onto the carrier and presented as a specific activity. The free enzyme showed the lowest specific hydrolytic—0.075 U/g protein and synthetic—9.999 U/g protein activities compared to immobilized biocatalysts. The enzyme immobilized onto untreated chokeberry pomace showed the highest specific hydrolytic activity (2.538 U/g protein). As the degree of purification of the pomace increased successively with hexane and ethanol, the activity of chokeberry pomace decreased. In contrast, apple pomace remained at a constant, small level, a level that is not significantly different from free lipase. Unprocessed apple pomace showed lower specific hydrolytic activity compared to chokeberry waste. This may be due to differences in the chemical composition and surface structure of the two types of carriers. Studies showed that chemical treatment of the carriers can alter their surface properties, affecting the enzyme activity. Hexane extraction can remove some lipids from the surface of the carriers, changing their properties and affecting enzyme–carrier interactions. In the case of chokeberry waste, hexane extraction lowered the specific activity, but it was still higher than for the free enzyme. Ethanol extraction, especially conducted in the Soxhlet apparatus, can remove various organic compounds (e.g., phenolics, sugars), which can change the surface properties of the carrier more dramatically, explaining the much lower enzyme activity after ethanol extraction [35]. The study by Girelli et al. [10] also showed better results than free lipase, demonstrating the importance of the immobilization process.

In the specific synthetic activity, the highest result was achieved for enzyme preparation immobilized onto native apple pomace (115 U/g protein) and was slightly lower in chokeberry pomace (77 U/g protein). Purification of the pomace led to a decrease in activity, as in the previous results. Perhaps the chemical surface area of the pomace influences the catalytic activity [36]. In the case of unprocessed carriers, natural components (e.g., lipids, polyphenols, sugars) can stabilize the enzyme structure, which is particularly important for enzymatic processes [37,38].

### 3.3. Substrate Specificity of Immobilized Lipases

**Figure 6:**
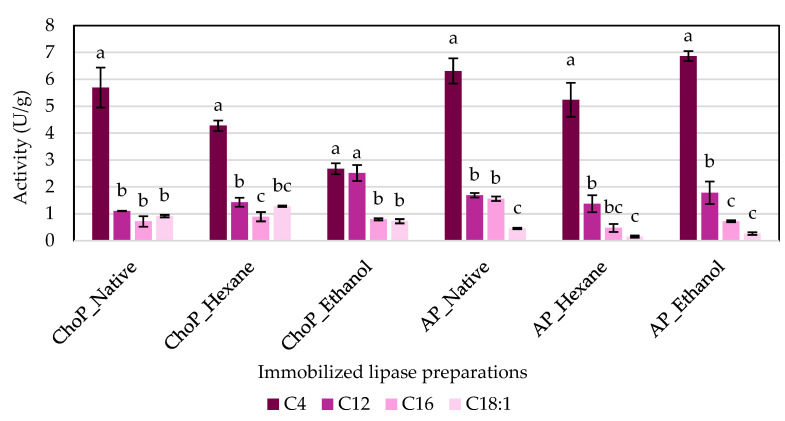
Comparison of hydrolytic activity of biocatalysts for the following substrates: *p*-nitrophenyl butyrate (C4:0), *p*-nitrophenyl laurate (C12:0), *p*-nitrophenyl palmitate (C16:0), and *p*-nitrophenyl oleate (C18:1). The means compared within one enzyme preparation, marked with different lowercase letters, are statistically different (α = 0.05). Abbreviations: ChoP—chokeberry pomace, AP—apple pomace.

Substrate specificity was analyzed for all the obtained biocatalysts (Figure 6). The four following esters were used in this experiment: *p*-nitrophenyl butyrate (C4:0), *p*-nitrophenyl laurate (C12:0), *p*-nitrophenyl palmitate (C16:0), and *p*-nitrophenyl oleate (C18:1). The results showed that the highest lipolytic activities were achieved against the substrate *p*-nitrophenyl butyrate (C4:0), where it was up to 7 U/g for lipase immobilized onto apple carrier purified with ethanol. The obtained biocatalysts also catalyzed reactions of other substrates (*p*-nitrophenyl laurate, palmitate, and oleate) but with lower activities. Similar observations were made by Druteika et al. [39], where a parental *Geobacillus* lipase GD-95 had the highest lipolytic activity with *p*-NP butyrate (C4:0) as a substrate. The lipase from *R. miehei* (RML) is known as a *sn*-1,3-specific lipase, whose natural function is the hydrolysis of triglycerides. Therefore, it is possible for them to catalyze hydrolysis reactions of both short-chain and long-chain fatty acid esters [15]. Our results confirmed that lipases immobilized onto apple and chokeberry pomaces are able to catalyze the hydrolysis reactions of *p*-nitrophenyl esters specifically.

### 3.4. Profile of pH Activity of Immobilized Lipase

**Figure 7:**
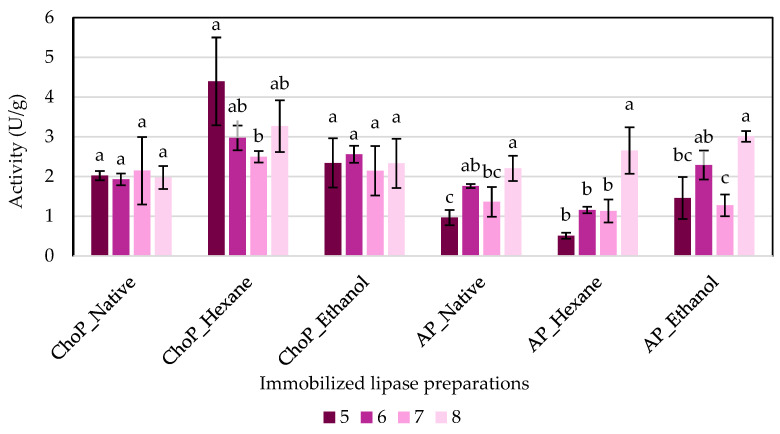
The effect of pH (ranging from 5 to 8) on the hydrolytic activity of the produced biocatalyst. The averages compared within a single enzyme preparation across the examined pH range, indicated by distinct lowercase letters, are significantly different (α = 0.05). Abbreviations: ChoP—chokeberry pomace, AP—apple pomace.

The impact of pH on the activity of lipase was also evaluated. Hydrogen ion concentration can affect the ability of lipases to catalyze various reactions, so hydrolytic activity was studied at pH in the range of 5 to 8 (Figure 7). Most of the biocatalysts immobilized onto chokeberry pomace were stable over the entire pH range, and their activities did not vary significantly. The exception is hexane-purified chokeberry preparations. In this case, the highest activity—4.4 U/g—was obtained for pH 5, and it was significantly higher compared to pH 7, where an activity of 2.5 U/g was obtained. The result suggests that lipases adsorbed on chokeberries had a more stable configuration and could be easily used as biocatalysts in reactions requiring varying pH conditions. Similar properties were investigated by Girelli et al. [10] and Jasinska et al. [34]. The researchers conducted a study on used coffee grounds in identical pH ranges and observed no clear differences between the study groups. Significantly different results were obtained for preparations immobilized onto apple pomace. In these groups, each purification method indicated independent groups that were significantly different depending on the pH value. In each group, the highest activity was obtained for tests with pH 8, which indicates that these are the proper conditions for biocatalysts immobilized onto apple pomace.

Takó et al. [40] reported that isolated and purified *R. miehei* lipase NRRL 5282 had a optimum pH between 6.8 and 7.4. The pH stability tests revealed that the purified lipase demonstrated significant stability, retaining over 70% of its activity within a pH range of 7.0 to 8.0. This indicates its classification as an alkaline-tolerant enzyme. Fé et al. [41] also found that free lipase RML (Palatase) showed the highest activity at pH 7. However, Zhang et al. [42] observed that pH 8 is optimum for RML immobilized onto the surface of *Saccharomyces cerevisiae* using whole-cell biocatalyst techniques. With this information and the data shown in the present paper, perhaps chokeberry pomace used as a support maintains the protective effect of the material, ensuring lipase stability in a wider range of pH compared with free enzymes. In contrast, apple pomace leads to higher activity of lipases in the alkaline zone.

### 3.5. The Recovery of Immobilized Biocatalysts

**Figure 8:**
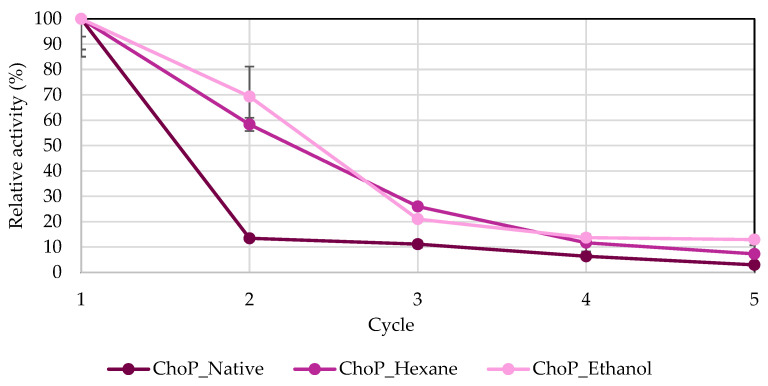
Recovery analysis of lipase immobilized onto chokeberry pomace. The highest hydrolytic activities of all biocatalysts were defined as 100%. Abbreviation: ChoP—chokeberry pomace.

**Figure 9:**
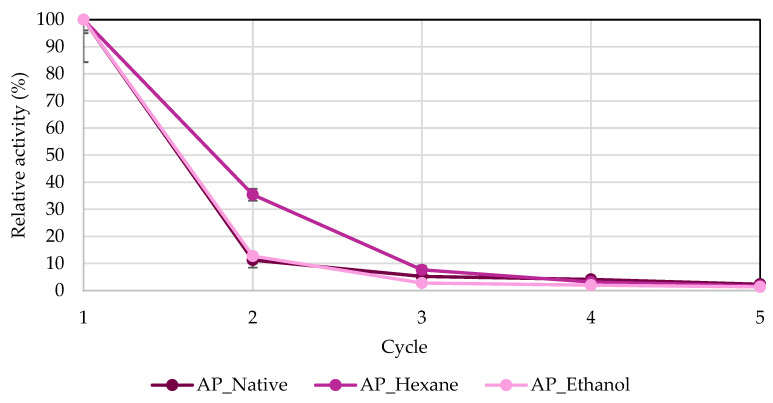
Recovery analysis of lipase immobilized onto apple pomace. The highest hydrolytic activities of all biocatalysts were defined as 100%. Abbreviation: AP—apple pomace.

Another important advantage of enzyme immobilization from an industrial standpoint is the ability to recover biocatalysts. Adsorption is a reversible method based on weak bonds, including van der Waals forces, hydrogen bonds, or hydrophobic interactions [8,34,43]. In the current research, lipase from *R. miehei* immobilized onto chokeberry and apple pomace was tested in five cycles. After each reaction, biocatalysts were retrieved, washed, and recycled (Figure 8 and Figure 9). Figure 8 describes the reusability of biocatalysts adsorbed on chokeberry pomace. It can be seen that the highest activity in each cycle, except for the third cycle, was for ethanol-treated chokeberry, while the lowest was for native chokeberry. The activity in the second cycle for hexane- and ethanol-treated chokeberries was lower by only 40 and 30%, respectively. Also, even in the fifth cycle, 5–10% activity was shown. These are promising observations considering that in the study presented by Karra-Châabouni et al. [44], where lipases were immobilized onto a cellulose carrier, *Rhizopus oryzae* lipase immobilized onto modified cellulose fibers yielded only 25% of its initial esterification activity after just three cycles. Activities of enzymes immobilized onto spent coffee grounds analyzed by Jasinska et al. [34] dropped by half as early as the second cycle, and after the fourth cycle, they were practically inactive. Similar observations to the abovementioned study were shown for apple pomaces, where the highest activity in the second cycle was observed for hexane-treated apple pomace, but it was less than 40%. Similarly, after the fourth cycle, the activities for all test groups ranged from 5–12%. The hydrophobic and electrostatic interactions can be affected by various immobilization factors, including pH levels, the amount and quality of reagents used, the type of enzyme, surface area, the chemical composition of the support, and ionic strength. Lignocellulosic waste shows promise as a support material due to the specific functional groups on its surface that can bond with lipases, as well as its considerable porosity. However, there is a risk of lipases leaching from the carrier, which may result in decreased activity during subsequent processing cycles [17,45].

## 4. Conclusions

This study examined reusing fruit waste as potential support for enzyme immobilization. The biocatalyst was microbial lipase from *R. miehei*, which was adsorbed on apple and chokeberry pomace, which are various and heterogeneous materials with different contents of cell walls. Their simple structure allowed lipase adsorption on the surface, resulting in the obtaining of lipolytic biocatalysts. The obtained immobilized enzyme preparations could catalyze the hydrolytic and synthetic reactions. It was observed that pretreating the fruit pomace before the immobilization process had a greater impact on the synthetic activity of biocatalysts than on hydrolytic activity, leading to a decrease in activity for samples with more purified pomaces (with removed lipids and polyphenols fractions).

The study showed how important is to choose the right carrier for lipase immobilization, as it can affect the properties of the biocatalyst. Although the lipase immobilized on native pomace had the highest synthetic activity, from an economic and environmental point of view, it is very beneficial since the waste obtained can be directly managed without purification. Further experiments showed that compounds contained in native pomace can potentially inhibit the activity of lipases. Moreover, higher activity in different pH ranges and the possibility of recovery in subsequent reaction cycles had biocatalysts immobilized on purified pomace (defatted and with removed polyphenols).

## Figures and Tables

**Table 1:** Cellulose, hemicellulose, and lignin content of supports—native apple and chokeberry pomaces.

Parameter	Unit	Native Apple Pomace	Native Chokeberry Pomace
CF—crude fiber	%DM	21.72 ± 0.21	24.45 ± 1.12
NDF		36.32 ± 0.06	55.16 ± 4.80
ADF		30.45 ± 0.13	51.63 ± 0.40
Cellulose (ADF-ADL)		20.99 ± 0.07	18.87 ± 0.32
Hemicellulose (NDF-ADF)		5.87 ± 0.97	3.53 ± 1.30
Total (CEL and HEM)		26.86 ± 0.13	22.40 ± 4.73
Lignin (ADL)		9.46 ± 0.20	32.76 ± 0.08

Abbreviations: CF—crude fiber, NDF—neutral detergent fiber, CEL—cellulose, HEM—hemicellulose, ADF—acid detergent fiber, ADL—acid detergent lignin, DM—dry mass.

## Data Availability

The data presented in this study are available on request from the corresponding authors (K.J. and B.Z.).
